# Lipopolysaccharide Binding Protein and Bactericidal/Permeability-Increasing Protein as Biomarkers for Invasive Pulmonary Aspergillosis

**DOI:** 10.3390/jof6040304

**Published:** 2020-11-20

**Authors:** Sigrid Bülow, Robert Heyd, Martina Toelge, Katharina U. Ederer, Annette Schweda, Stefan H. Blaas, Okka W. Hamer, Andreas Hiergeist, Jürgen J. Wenzel, André Gessner

**Affiliations:** 1 Institute of Clinical Microbiology and Hygiene, University Hospital Regensburg, Franz-Josef-Strauß-Allee 11, 93053 Regensburg, Germany; robert.heyd@ukr.de (R.H.); martina.toelge@ukr.de (M.T.); katharina.ederer@ukr.de (K.U.E.); andreas.hiergeist@ukr.de (A.H.); juergen.wenzel@ukr.de (J.J.W.); 2 Department of Pneumology, Klinik Donaustauf, Ludwigstraße 68, 93093 Donaustauf, Germany; annette.schweda@ukr.de (A.S.); stefan.blaas@ukr.de (S.H.B.); 3 Department of Radiology, University Hospital Regensburg, Franz-Josef-Strauß-Allee 11, 93053 Regensburg, Germany; okka.hamer@ukr.de; 4 Department of Radiology, Klinik Donaustauf, Ludwigstraße 68, 93093 Donaustauf, Germany

**Keywords:** lipopolysaccharide binding protein, bactericidal/permeability-increasing protein, interleukin-8, *Aspergillus*, invasive pulmonary aspergillosis, bronchoalveolar lavage, galactomannan, biomarker

## Abstract

Early diagnosis of invasive pulmonary aspergillosis (IPA) is crucial to prevent lethal disease in immunocompromized hosts. So far, lipopolysaccharide binding protein (LBP) and bactericidal/permeability-increasing protein (BPI) levels have not been evaluated as biomarkers for IPA. IL-8, previously introduced as a biomarker for IPA, was also included in this study. Bronchoalveolar lavage fluid (BALF) of IPA patients and control patients with non-infectious lung disease was collected according to clinical indications. Measurements in BALF displayed significantly higher levels of LBP (*p* < 0.0001), BPI (*p* = 0.0002) and IL-8 (*p* < 0.0001) in IPA compared to control patients. Receiver operating characteristic curve analysis revealed higher AUC for LBP (0.98, 95% CI 0.95–1.00) than BPI (0.84, 95% CI 0.70–0.97; *p* = 0.0301). Although not significantly different, AUC of IL-8 (0.93, 95% CI 0.85–1.00) also tended to be higher than AUC for BPI (*p* = 0.0624). When the subgroup of non-hematological patients was analyzed, test performance of LBP (AUC 0.99, 95% CI 0.97–1.00), BPI (AUC 0.97, 95% CI 0.91–1.00) and IL-8 (AUC 0.96, 95% CI: 0.90–1.00) converged. In conclusion, LBP and—to a lesser extend—BPI displayed high AUCs that were comparable to those of IL-8 for diagnosis of IPA in BALF. Further investigations are worthwhile, especially in non-hematological patients in whom sensitive biomarkers for IPA are lacking.

## 1. Introduction

Invasive *Aspergillus* infection, most frequently manifesting as invasive pulmonary aspergillosis (IPA), is an emerging disease in immunocompromized hosts worldwide, affecting around 200,000 patients per year [1,2]. Mortality rates ranging between 35.3% and 77.8% are of special concern in both hematological patients, i.e., patients with hematologic malignancies and hematopoietic stem cells transplantation, and non-hematological patients [2,3,4,5]. To improve clinical outcome, early diagnosis resulting in immediate systemic antifungal therapy is crucial [6]. However, mycological diagnosis of IPA is challenging since routinely available diagnostic tests in serum or respiratory specimens lack sufficient accuracy. Cultures or *Aspergillus*-specific PCR of bronchoalveolar lavage fluid (BALF) display limited sensitivities of 19–50% and 44–70%, respectively [6,7]. Thus, fungal biomarkers, such as galactomannan (GM) and (1,3)-β-d-glucan, have been extensively studied. However, serum GM has moderate sensitivity and specificity of 71–73.5% and 83.5–89%, respectively, when a cut-off of 0.5 is used [8,9]. Importantly, these values strongly differ depending on the patient population addressed [8]. Although test sensitivity markedly increases in hematologic patients by implementation of serial testing [10,11,12], sensitivity rates are beneath 30% in patients receiving mold-active antifungal prophylaxis, in pediatric populations and in non-neutropenic patients, such as lung transplant recipients [13,14,15,16]. Testing of GM in BALF reveals higher sensitivity compared to GM in serum of neutropenic and non-neutropenic patients [16], but vary between 50% and 92% with corresponding specificities between 73% to 98% [14,17,18,19]. Collectively, improved markers to diagnose invasive mold infections in hematological and non-hematological patients are urgently needed.

Immunological markers have the potential to contribute to the diagnosis of IPA [20]. Recently, interleukin (IL)-8 was described as the most sensitive biomarker in serum and BALF of hematological patients from a screen of more than 32 cytokines and chemokines [21]. Prospective follow-up studies in hematological patients with suspected pulmonary infection confirmed sensitivities of up to 82% in serum, and 91% in BALF for diagnosis of IPA [22,23]. Similarly to IL-8, the two members of the tubular lipid binding proteins (TULIP) family [24] lipopolysaccharide binding protein (LBP) and bactericidal/permeability-increasing protein (BPI) are increased in bacterial infections [25,26,27,28,29,30]. However, their role in *Aspergillus* infection has not yet been determined thus far. In our study, performance of LBP was high in both hematological and non-hematological patients, whereas performance of BPI approximated LBP in non-hematological patients. Therefore, LBP and BPI in BALF are promising biomarkers for IPA.

## 2. Materials and Methods

Patient selection–In the IPA group, patients undergoing bronchoscopy between January 2013 and December 2019 at the University Hospital Regensburg, Germany, and fulfilling the inclusion criteria, were retrospectively enrolled. BALF and serum samples were collected for routine testing according to clinical indications. Inclusion criteria were (I) patients older than 18 years in whom (II) serum and BALF were routinely drawn within 10 days, and GM was thereby positive in both serum and BALF, plus (III) left-over samples of the BALF were available. All patient records were evaluated for their predisposing risk of developing IPA, including hematological disease and varying predisposing morbidities such as solid organ transplantation, solid tumors, autoimmune diseases, chronic respiratory diseases, recent sepsis episodes, liver cirrhosis as well as severe influenza or other viral infections [3,16,31]. 18 of 19 patients received high-resolution computed tomography (CT) scans (i.e., slice thickness 1 mm), whereas in one patient the CT scan performed had a slice thickness of 5 mm. CT scans were evaluated by a thoracic radiologist (20 years experience) for the presence of typical morphological criteria according to the recently revised guidelines of the European Organization for Research and Treatment of Cancer Invasive Fungal Infections Cooperative Group and the Mycoses Study Group of the National Institute of Allergy and Infectious Disease (EORTC-MSG criteria) [32]. 18 of 19 enrolled patients met these radiological EORTC-MSG criteria for IPA. The remaining patient who suffered from emphysema did not show any nodules, with or without halo, air-crescent sign, cavity, wedge-shaped, segmental or lobar consolidation but displayed radiological findings compatible with IPA, namely disseminated areal consolidations and diffuse ground glass opacities [33,34]. Adult patients with interstitial lung disease (ILD) of non-infectious origin, who were hospitalized in an associated hospital in Donaustauf, Germany, and in whom BALF and serum samples were collected for routine testing following clinical indications, were used as age- and sex-matched control group. Exclusion criteria for both the IPA and control group included concurrent bacterial pulmonary infections or systemic infection of non-*Aspergillus* origin. No control patients had concurrent possible, probable or proven pulmonary *Aspergillus* infection.

Measurement of galactomannan–GM detection in serum and BALF was performed using Platelia *Aspergillus* ELISA (Bio-Rad Laboratories, Marnes-la-Coquette, France), and was determined in the diagnostic laboratory of the Institute of Clinical Microbiology and Hygiene, Regensburg. Samples exceeding the index of the positive control were frozen at −20 °C and titrated alongside with measurements of BPI, LBP, myeloperoxidase (MPO) and cytokines. According to Gonçalves et al. [21] cut-off points of 0.5 were applied for GM indices of both serum and BALF since 68.4% of the patients received mold-active antifungals at the time of bronchoscopy. BALF samples were initially stored at 4 °C until routine testing was finalized, and then subsequently transferred to −20 °C for storage.

Measurement of BPI, LBP, MPO and cytokines in BALF–All available leftover samples of BALF stored at the Institute of Clinical Microbiology and Hygiene, University Hospital Regensburg tested positive for GM in serum and BALF were analyzed. All samples were pseudonymized, and clinical data were blinded to the test performer. BPI, LBP, MPO, IL-1β and IL-8 were determined using Luminex^®^ technology (Austin, TX, USA) with lower limits of quantification of 1.4 ng/mL for BPI, 0.5 ng/mL for LBP, 0.2 ng/mL for MPO, 6.0 pg/mL for IL-1β and 2.2 pg/mL for IL-8. BPI and LBP were measured using specific antibody pairs (anti-BPI capture antibody 3F9 and anti-BPI detection antibody 4H5 (Hycult Biotech, Uden, The Netherlands); anti-LBP capture antibody biG43 and anti-LBP detection antibody biG412 (Biometec, Greifswald, Germany)). Biotinylation of the detection antibodies was performed using the Lightning-Link^®^ Biotin Conjugation Kit (Innova Biosciences, Cambridge, UK). MPO, IL-1β and IL-8 were determined by the ProcartaPlex^®^ Multiplex Immunoassay (eBioscience, Santa Clara, CA, USA).

Ethics Statement–This study was carried out in accordance with the recommendations of the Declaration of Helsinki. Diagnostic leftover samples stored at the Institute of Clinical Microbiology and Hygiene, University Hospital Regensburg were used for BALF analysis. The protocol was approved by the local ethics committee (Ethikkommision an der Universität Regensburg, EC-No. 18-1269-101, 2016/11/16 and 10-101-0078, 2010/06/25).

Statistical analysis–Relation of clinical data and biomarkers were unblinded for statistical testing. Analyses were performed using GraphPad Prism, version 7.01 (GraphPad Software, San Diego, CA, USA) and R, version 4.0.2 (The R Foundation for Statistical Computing, Vienna, Austria). Results are depicted as means ± SEM. Statistical tests were performed as described in the figure legends. *P*-values < 0.05 were considered statistically significant. Receiver operating characteristic (ROC) curve analysis was performed and the area under the curve (AUC) value was calculated including the 95% confidence interval (95% CI) by using the ci() function of the pROC package version 1.16.2 in R [35]. DeLong’s test for two correlated ROC curves was used to compare the AUC of two ROC curves as implemented in the pROC package. Principal coordinates analysis (PCoA) was used to determine clustering of IPA subgroups and control groups. The underlying distance matrix was created by calculating euclidean distances between samples after log-transformation of the dataset. Significant differences between subgroups were analyzed by permutational multivariate analysis of variance applying the pairwiseAdonis package. Resulting *p*-values were adjusted for multiple testing by the Bonferroni correction method.

## 3. Results

**Figure 1:**
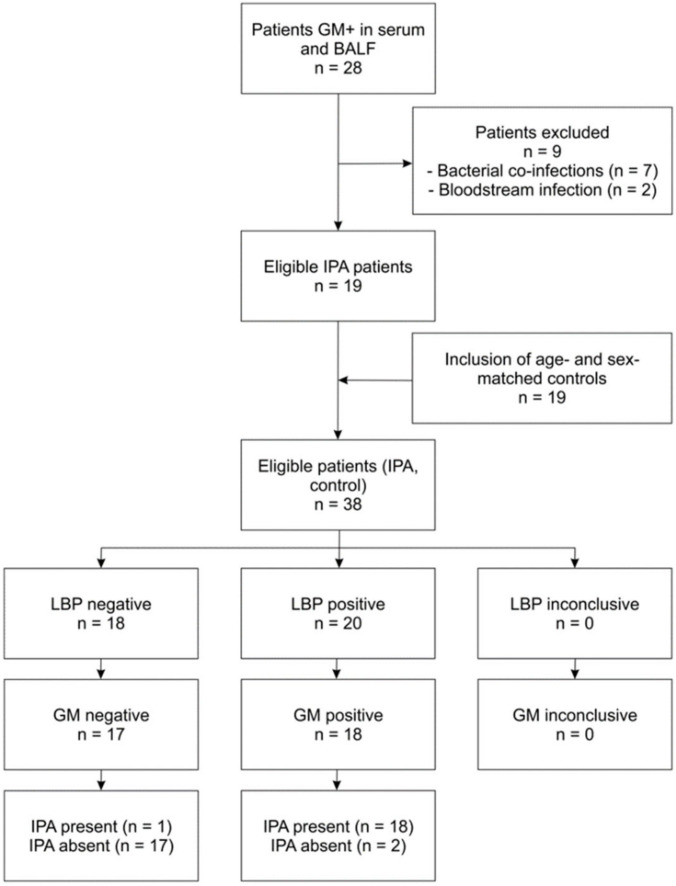
Flowchart indicating selection of patients and performance. The chart is exemplified for LBP in BALF as the index test, and GM in BALF as the reference test. The test positivity cut-off point of 18.9 pg/mL for LBP was determined according to the results of the ROC curve analysis depicted in Figure 2. Compared to GM, LBP displayed a sensitivity of 94.7%, a specificity of 84.2%, a positive predictive value (PPV) of 0.90 and a negative predictive value (NPV) of 0.94 in the selected patient group.

Patients were selected as described in the methods and displayed in a respective flowchart (Figure 1). Patient characteristics are summarized in Table 1 (IPA patients) and Table S1 (age- and sex-matched control patients).

**Figure 2:**
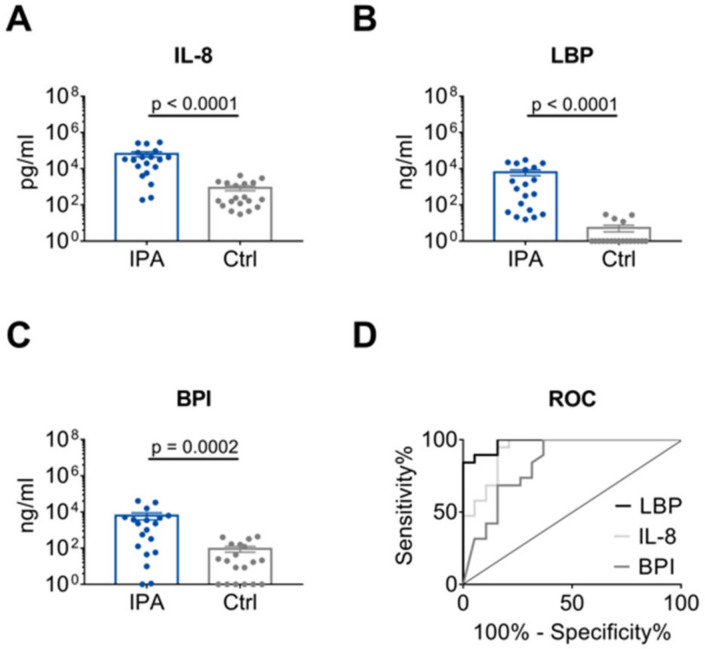
Distinction of IPA and Ctrl patients by IL-8, LBP and BPI. (**A**–**C**) Levels of IL-8, LBP and BPI in the BALF of 19 patients with IPA compared with 19 age- and sex-matched Ctrl patients. Data are presented as mean ± SEM. *P*-values were determined by Mann-Whitney U test. (**D**) AUC as determined by ROC curve analysis for IL-8, LBP and BPI values depicted in (**A**–**C**) for IPA and Crtl patients.

A significant difference in IL-8, LBP and BPI level was found between IPA and control patients (Figure 2A–C). According to ROC curve analysis (Figure 2D, Table 2), discrimination between the IPA and control group was high for LBP and IL-8 with AUCs of 0.98 (95% CI 0.95–1.00) and 0.93 (95% CI 0.85–1.00), respectively. In contrast, AUC for BPI (0.84, 95% CI 0.70–0.97) was significantly lower than AUC for LBP (*p* = 0.0301), and also tended to be lower than AUC for IL-8 (*p* = 0.0624). Since BPI and MPO are both derived from azurophil granula of neutrophils, MPO was also analyzed displaying an AUC of 0.84 (95% CI 0.69–0.98; Figure S1, Table 2). Furthermore, AUC for IL-1β, a cytokine derived from multiple cell types, was quantified (0.81, 95% CI 0.67–0.96; Figure S1, Table 2).

**Figure 3:**
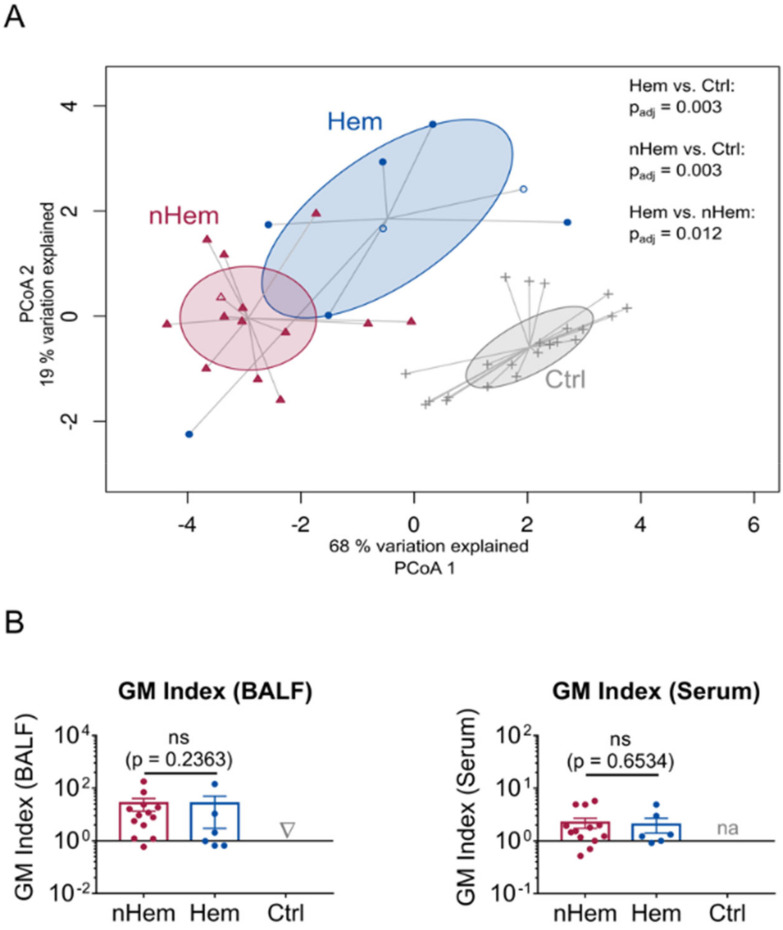
Comparison of tested biomarkers in the subgroup of nHem and Hem patients. (**A**) PCoA including CRP, WBC, GM in BALF, IL-8, IL-1β, LBP, BPI and MPO. Filled red symbols indicate nHem patients (*n* = 13), filled blue symbols Hem patients (*n* = 6) and grey symbols patients of the control group (Ctrl; *n* = 19). Patients not meeting inclusion criteria, because serum GM was beneath the cut-off point despite typical radiological findings, are marked as non-filled symbols (*n* = 3). Differences between subgroups were analyzed by permutational multivariate analysis of variance. *P*-values adjusted for multiple testing by the Bonferroni correction method are indicated (p adj). (**B**) GM in BALF and serum are compared between nHem (*n* = 13), Hem patients (*n* = 6) and control group (*n* = 19). Data are presented as mean ± SEM and *p*-values were determined by Mann-Whitney *U* test, non-significant (ns) results are marked. GM of serum in the control group was not analyzed (na).

New biomarkers are particularly needed in the non-hematological patient group since, as stated previously, conventional diagnostic tests are generally of limited sensitivity and specificity in this population. Thus, the subgroups of hematological (Hem) and non-hematological (nHem) IPA patients were compared by PCoA, revealing significantly distinct clustering of hematological patients (Figure 3A). Interestingly, three patients fulfilling the EORTC-MSG criteria for IPA [32], but not meeting our strict inclusion criteria due to negative serum clustered within the hematological and non-hematological subgroup according to their underlying disease (Figure 3A and Figure S2). As expected, WBC was significantly lower in the hematological subgroup than in the non-hematological subgroup, whereas CRP as well as GM in serum and BALF did not differ (Figure 3B and Figure S3). When clinical parameters were compared, hematological patients showed better IPA outcome with increased survival, a higher rate of systemic corticosteroid intake and a higher rate of mold-active therapy started at least 2 days prior to bronchoalveolar lavage (Figure S4).

**Figure 4:**
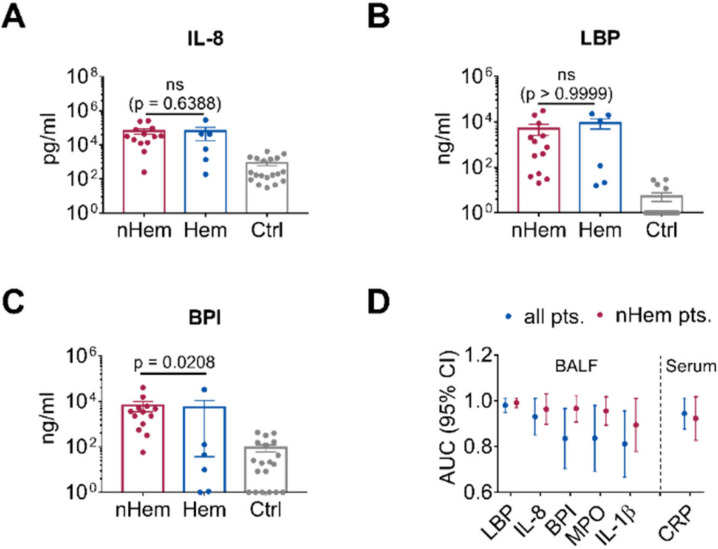
Discrimination between nHem, Hem and Ctrl patients when using different biomarkers. (**A**–**C**) Comparison of IL-8, LBP and BPI levels in the BALF of nHem (*n* = 13) and Hem patients with IPA (*n* = 6) as opposed to the control patients (*n* = 19). Data are presented as mean ± SEM and *p*-values were determined by Mann-Whitney U test, non-significant (ns) results are marked. (**D**) Comparison of AUC of cytokines including IL-1β and MPO in BALF and CRP in serum as evaluated in ROC curve analysis for all IPA patients and those IPA patients with an underlying non-hematological disease including 95% CI.

When biomarkers of hematological patients and non-hematological patients were compared, levels of LBP and IL-8 were not significantly different between the subgroups (Figure 4A,B). However, in accordance with the lower WBC, the neutrophil-derived marker BPI was significantly lower in the hematological patients (*p* = 0.0208; Figure 4C). ROC curve analysis of the non-hematological patients revealed high AUC for LBP (0.99, 95% CI 0.97–1.00), BPI (0.97, 95% 0.91–1.00) and IL-8 (0.96, 95% CI 0.90–1.00; Figure S5C and Table 3). Again, AUC of MPO (0.96, 95% CI 0.90–1.00) and IL-1β (0.89, 95% CI 0.78–1.00) were not higher than those for BPI or the other markers tested (Figure S5 and Table 3). Direct comparison showed high AUC for LBP, with low variability in all IPA patients and in the non-hematological subgroup. Comparable performance of IL-8 and BPI was found in the non-hematological subgroup (Figure 4D and Table 3).

## 4. Discussion

Here we describe BPI and LBP as potential biomarkers in the diagnosis of IPA. Although BPI has been known to increase in Gram-negative and Gram-positive infections, such as sepsis or bacterial meningitis [25,26,36], and LBP is described as a marker in bacterial as well as a few cases of *Candida* sepsis [27,28,29,30], to date neither BPI nor LBP have been published as biomarkers in *Aspergillus* infections. As shown by calculating AUC, LBP displays high performance in diagnosing IPA in the studied cohort.

In line with previous reports on hematological risk patients [21,22,23], we can also confirm IL-8 as a potential biomarker in a cohort also containing non-hematological patients, and further implement the utility and robustness of IL-8 measurement. Whereas IL-8 is mainly derived from the bronchopulmonary epithelium and antigen-presenting cells, such as alveolar macrophages [15], LBP mainly originates from hepatocytes [37,38], which might suggest induction via circulating fungal antigen after angioinvasion by *Aspergillus* hyphae or via systemic cytokine response. However, respiratory epithelial cells, known targets of *A. fumigatus* hyphae [39], were also shown to express LBP [40]. Although we did not compare serum LBP to serum GM, we speculate that LBP level is more sustained than the transient increase in GM, which is rapidly cleared from circulation by neutrophils, kidney and liver [41]. In line with the presence of neutropenic patients in our IPA group, AUC of neutrophil-derived BPI was lower compared to LBP. Performance of this neutrophil marker increased when non-hematologic IPA patients were exclusively addressed. In this cohort, BPI was similar to IL-8 as measured by AUC.

This study was performed retrospectively with a stringent selection of patients attributed to the lack of a gold standard for diagnosis of IPA. Inclusion of patients with positive GM in serum and BALF might preferentially include patients with more severe involvement. Indeed, high mortality occurred especially in non-hematological patients. This subgroup included a high proportion of IPA patients receiving ECMO, a risk group with described mortality rates of 80.0% [42]. Thus, in a less selected cohort, test performance might differ and would also circumvent limitation due to the small number of eligible patients. Although patients with chronic respiratory disease, especially when on steroids or other immunosuppressive therapies [3,32], are at risk for IPA, a more diverse control group might have been preferable. Nevertheless, by referring to IL-8 as a threshold, LBP turned out to be a very interesting candidate as an immunological biomarker in invasive aspergillosis. As already stated, BPI seems to be eligible for non-hematological patients without neutropenia. Since performance of GM testing in non-hematological patients is lower than in hematological patients [2,8], testing for LBP and/or BPI might be of special value in this patient group.

As stated, LBP and BPI levels are known to be increased in bacterial bloodstream infections [25,26,27,28,29,30]. Although the authors are not aware of any specific data in the literature, respiratory co-infections with bacteria might also cause elevation of LBP or BPI in BALF. Therefore, seven patients with concurrent pulmonary bacterial infections, and two patients with non-*Aspergillus* sepsis have been excluded from this study in order to examine biomarkers in IPA without bacterial co-infections. Congruently, no bacterial co-infections were present in the control group either. Thus, the question remains open whether bacterial, viral and non-*Aspergillus* fungal infections might influence specificity of the test and cause false-positive results. Conversely, fungal biomarkers are lacking in mucormycosis [43] and elevation of immunological markers such as LBP or BPI could possibly indicate the need for extended testing, such as Mucorales-specific PCR. Thus far, a combination with fungus-specific markers will most likely be necessary for initial diagnosis of IPA (add-on test), but might not be essential for other indications, such as monitoring of therapy response, detection of breakthrough invasive fungal infections, de-escalation or withholding of therapy and prediction of clinical outcome once diagnosis is established. Blood samples were not evaluated in this study, but might broaden the applications of LBP or BPI and avoid invasive bronchoalveolar lavage. Additionally, evaluation might also be worthwhile in extrapulmonary *Aspergillus* manifestations such as the central nervous system [44].

In our study, *Aspergillus* superinfections were present in three patients with influenza pneumonia, and one patient with pneumonia caused by respiratory syncytial virus. Importantly, association of viral pneumonia and IPA is not only common in influenza [45,46,47], but is also described in COVID-19 patients [48,49,50,51]. Interestingly, LBP levels were elevated in serum of patients with severe versus non-severe SARS-CoV-2 infection in an unbiased proteomic screen [52], possibly indicating fungal superinfection when interpreted within the context of our data. Screening of LBP levels might therefore contribute to early diagnosis of IPA in the case of severe viral pneumonia. Systemic and local co-infections were frequent in our cohort (7.1% and 25.0%, respectively), which may be attributed to the specific selection criteria or the accumulation of complicated cases at the University Hospital Regensburg. Studies with larger cohorts are needed to evaluate the frequency and relevance of bacterial (co-)infections in IPA patients, as well as patients at risk for IPA, regarding not only the accuracy of biomarkers, but also the impact on severity of IPA. In conclusion, the first description of LBP and BPI as potential biomarkers for diagnosing IPA in this study warrants further evaluation in a prospective setting.

## Figures and Tables

**Table 1:** Characteristics of IPA patients.

Patient Characteristics		
Sex	Female Male	5 (26.3%) 14 (73.7%)
Age (in years)	Median (Range)	55 (28–81)
Disease	Hematological Non-hematological	6 (31.6%) 13 (68.4%)
ECMO	Total Hematological patients Non-hematological patients	5 (26.3%) 0 5
Mortality	Total	9 (47.4%)
Systemic treatment with corticosteroids	Total	10 (52.6%)
Mold-active antifungal prophylaxis/treatment (≥2 days before sampling)	Total	13 (68.4%)
**Laboratory Results**		
GM in serum	Mean ± SD	2.2 ± 1.7
GM in BALF	Mean ± SD	26.7 ± 48.9
WBC (1000/μL)	Mean ± SD	15.9 ± 14.6
CRP (mg/L)	Mean ± SD	126.6 ± 111.3
Relevant bacterial pathogens in BALF	PCR and/or culture (*Klebsiella pneumoniae*, *Legionella pneumophila*, *Mycobacterium tuberculosis*, *Staphylococcus aureus*, *Stenotrophomonas maltophilia*)	7 (excluded)
Relevant pathogens in blood culture	Culture (*Staphylococcus aureus*, *Candida glabrata)*	2 (excluded)
*Pneumocystis* in BALF	PCR	1 (5.3%)
Mucorales in BALF	PCR and/or culture	2 (10.5%)
Herpesviridae within 14 days of sampling	PCR for HSV, EBV, CMV, HHV-6	3 (15.8%)
**Patient Subgroups**		
Hematological subgroup	Total Acute myeloid leukemia Acute lymphoblastic leukemia Chronic lymphocytic leukemia Non-Hodgkin lymphoma	6 (100.0%) 3 (50.0%) 1 (16.7%) 1 (16.7%) 1 (16.7%)
	Allogenic hematopoietic stem cell transplantations	3 (50.0%)
	Graft-versus-host disease	2 (33.3%)
Non-hematological subgroup	Total Solid organ transplantation Solid tumor Chronic respiratory disease Liver cirrhosis Recent sepsis episode Severe influenza ^1^ Other viral infections ^2^	13 (100.0%) 2 (15.4%) 1 (7.7%) 2 (15.4%) 2 (15.4%) 3 (23.1%) 4 (30.8%) 1 (7.7%)

^1^ Concurrent COPD, ^2^ Respiratory syncytial virus infection in a patient with renal transplantation. Abbreviations: CMV (cytomegalovirus); CRP (C-reactive protein); EBV (Epstein–Barr virus); ECMO (extracorporeal membrane oxygenation), HHV-6 (human herpesvirus 6); HSV (herpes simplex virus); PCR (polymerase chain reaction); WBC (white blood count).

**Table 2:** ROC curve analysis for all IPA patients.

Biomarker	AUC	95% CI	*p*-Value (ref. LBP)	*p*-Value (ref. IL-8)
LBP	0.98	0.95–1.00	-	ns (0.2309)
IL-8	0.93	0.85–1.00	ns (0.2309)	-
BPI	0.84	0.70–0.97	0.0301	ns (0.0624)
MPO	0.84	0.69–0.98	ns (0.0501)	ns (0.0787)
IL-1β	0.81	0.67–0.96	0.0263	0.0429

ROC curves for LBP and IL-8 were compared as indicated by DeLong’s test for two correlated ROC curves. Abbreviation: ref. reference protein.

**Table 3:** ROC curve analysis for nHem IPA patients.

Biomarker	AUC	95% CI
LBP	0.99	0.97–1.00
IL-8	0.96	0.90–1.00
BPI	0.97	0.91–1.00
MPO	0.96	0.90–1.00
IL-1β	0.89	0.78–1.00

ROC curves for the indicated biomarkers were compared by DeLong’s test for two correlated ROC curves and showed no significant difference.

## Supplementary Materials

The following are available online at https://www.mdpi.com/2309-608X/6/4/304/s1, Figure S1: Distinction of IPA and Ctrl patients by MPO and IL-1β. Figure S2: Comparison of IL-8, LBP, BPI, MPO and IL-1β. Figure S3: Comparison of tested biomarkers in the subgroup of nHem and Hem patients. Figure S4: Comparison of clinical parameters in the subgroup of nHem and Hem patients. Figure S5: Discrimination between nHem and Hem patients with IPA and Crtl patients regarding MPO and IL-1. Table S1: Characteristics of control patients.
