# Supplementary material for: Lipopolysaccharide Binding Protein and Bactericidal/Permeability-Increasing Protein as Biomarkers for Invasive Pulmonary Aspergillosis
Source: J Fungi (Basel). 2020 Nov 20;6(4):304. doi: 10.3390/jof6040304 (PMC7712449; doi:10.3390/jof6040304)
Supplement: Supplementary file 1 [file jof-06-00304-s001.pdf]

Supplementary Materials

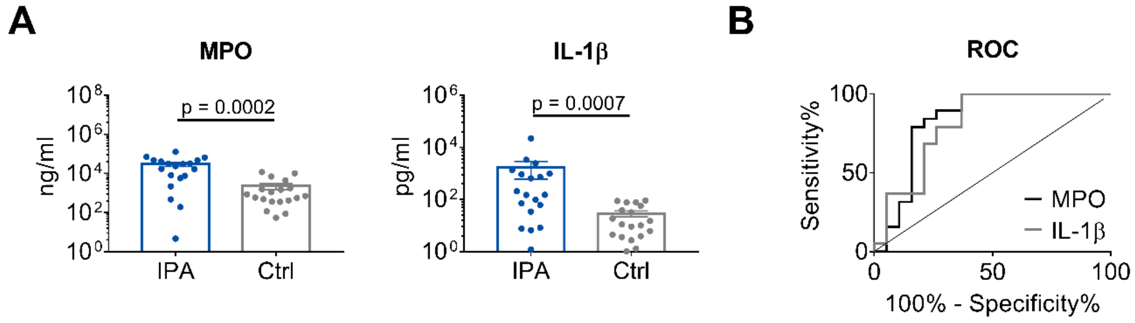

Figure S1. Distinction of IPA and Ctrl patients by MPO and IL-1β. **(A)** Levels of MPO and IL-1β in the BALF of 19 patients with IPA compared with 19 age- and sex-matched Ctrl patients. Data are presented as mean ± SEM. *P*-values were determined by Mann-Whitney *U* test. **(B)** Area under the curve (AUC) as determined by ROC curve analysis for MPO and IL-1β values depicted in (A) for IPA and Ctrl patients.

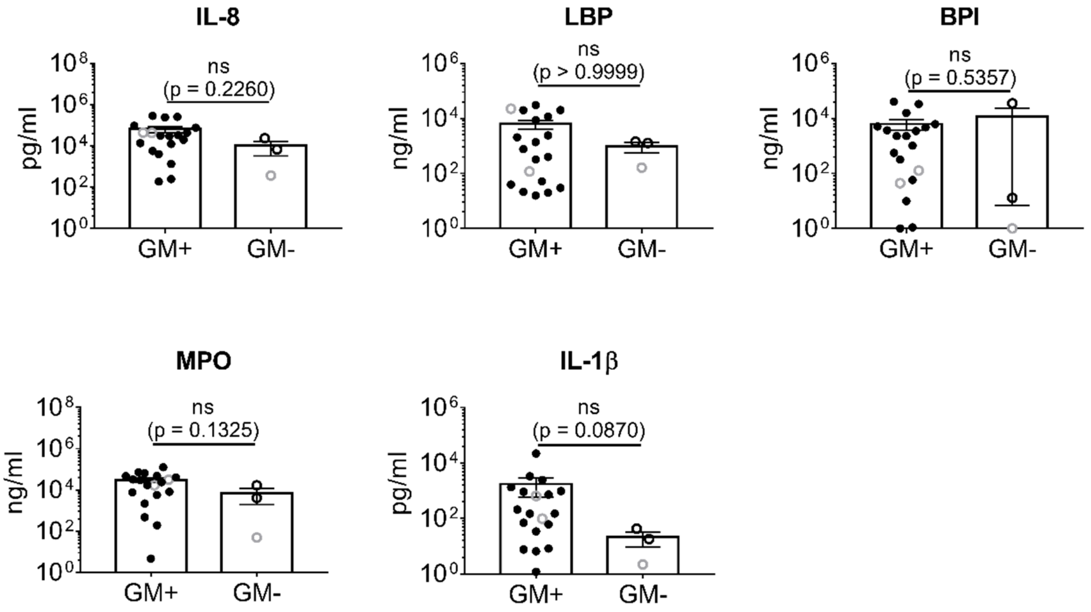

Figure S2. Comparison of IL-8, LBP, BPI, MPO and IL-1β. Comparison of included patients (GM positive in serum and BALF, GM+), and patients not meeting inclusion criteria because serum GM was beneath the cut-off, despite typical radiological findings (GM positive in serum, but negative in BALF, GM-). Patients with Mucorales co-infection are marked in grey, non-filled symbols. Data are presented as mean ± SEM. Mann-Whitney *U* test was used for the statistical analysis, non-significant (ns) results are marked.

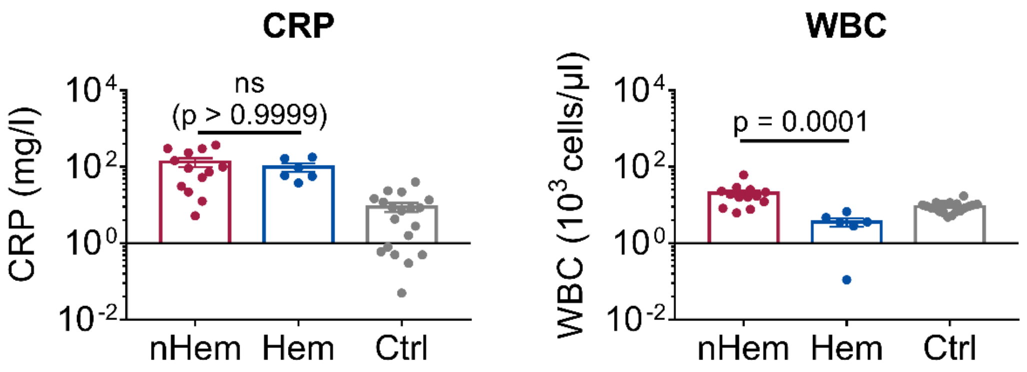

Figure S3. Comparison of tested biomarkers in the subgroup of nHem and Hem patients. CRP in serum and WBC are compared between nHem (n = 13), Hem (n = 6) and Ctrl group (n = 19). Data are presented as mean ± SEM and p-values were determined by Mann-Whitney U test, non-significant (ns) results are marked.

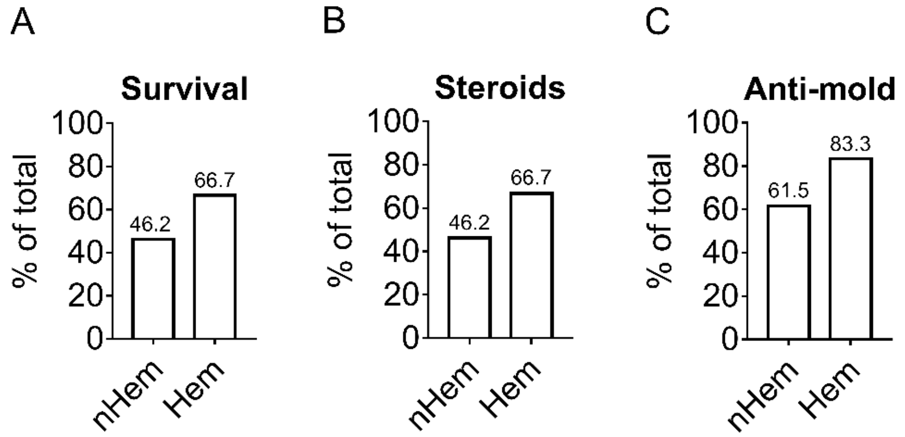

Figure S4. Comparison of clinical parameters in the subgroup of nHem and Hem patients. Survival **(A)**, use of systemic steroids at the time point when BALF was obtained **(B)** and anti-mold therapy for more than 2 days prior to the performance of the bronchoalveolar lavage **(C)** are depicted.

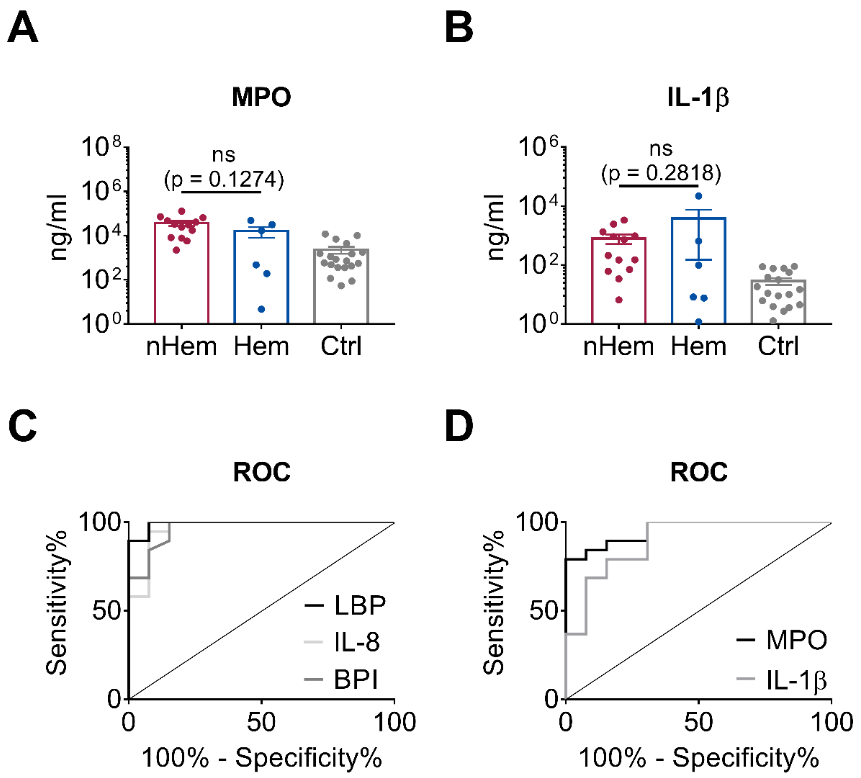

Figure S5. Discrimination between nHem and Hem patients with IPA and Crtl patients regarding MPO and IL-1β. (A, B) Comparison of MPO and IL-1β levels in the BALF of nHem (n = 13) and Hem patients with IPA (n = 6) as opposed to the Ctrl patients (n = 19). Data are presented as mean ± SEM. Mann-Whitney U test was used for the statistical analysis, non-significant (ns) results are marked. (C, D) ROC for IL-8, LBP, BPI as well as MPO and IL-1β in BALF for nHem versus Crtl patients.

Table S1. Characteristics of control patients.

Patient Characteristics		
Sex	Female	5 (26.3%)
	Male	14 (73.7%)
Age (in years)	Median (Range)	57 (28–81)
Idiopathic interstitial pneumonia	Total	7 (36.8%)
	Usual interstitial pneumonia	3 (15.8%)
	Non-specific interstitial pneumonia	2 (10.5%)
	Desquamative interstitial pneumonia	1 (5.3%)
	Cryptogenic organizing pneumonia	1 (5.3%)
Hypersensitivity pneumonitis		6 (31.6%)
Sarcoid		3 (15.8%)
ILD unclassified		3 (15.8%)
Mortality		none
Systemic treatment with corticosteroids		8 (42.1%)
Mold-active antifungal prophylaxis/treatment (≥2 days before sampling)		none
**Laboratory Results**		
GM in serum	Mean ± SD	not analyzed
GM in BALF	Mean ± SD	0.2 ± 0.1
WBC (1000/µL)	Mean ± SD	9.1 ± 2.8
CRP (mg/L)	Mean ± SD	8.9 ± 10.2
Relevant bacterial pathogens in BALF	PCR and/or culture	none
